# Non-triggered quantification of central and peripheral pulse-wave velocity

**DOI:** 10.1186/1532-429X-13-81

**Published:** 2011-12-21

**Authors:** Michael C Langham, Cheng Li, Felix W Wehrli

**Affiliations:** 1 Department of Radiology, University of Pennsylvania Medical Center, 3400 Spruced Street, Philadelphia, (19104), USA

## Abstract

**Purpose:**

Stiffening of the arteries results in increased pulse-wave velocity (PWV), the propagation velocity of the blood. Elevated aortic PWV has been shown to correlate with aging and atherosclerotic alterations. We extended a previous non-triggered projection-based cardiovascular MR method and demonstrate its feasibility by mapping the PWV of the aortic arch, thoraco-abdominal aorta and iliofemoral arteries in a cohort of healthy adults.

**Materials and Methods:**

The proposed method "simultaneously" excites and collects a series of velocity-encoded projections at two arterial segments to estimate the wave-front velocity, which inherently probes the high-frequency component of the dynamic vessel wall modulus in response to oscillatory pressure waves. The regional PWVs were quantified in a small pilot study in healthy subjects (N = 10, age range 23 to 68 yrs) at 3T.

**Results:**

The projection-based method successfully time-resolved regional PWVs for 8-10 cardiac cycles without gating and demonstrated the feasibility of monitoring beat-to-beat changes in PWV resulting from heart rate irregularities. For dul-slice excitation the aliasing was negligible and did not interfere with PWV quantification. The aortic arch and thoracoabdominal aorta PWV were positively correlated with age (p < 0.05), consistent with previous reports. On the other hand, the PWV of the iliofemoral arteries showed decreasing trend with age, which has been associated with the weakening of muscular arteries, a natural aging process.

**Conclusion:**

The PWV map of the arterial tree from ascending aorta to femoral arteries may provide additional insight into pathophysiology of vascular aging and atherosclerosis.

## Background

In elastic arteries, e.g. aorta and carotid arteries, the repeated mechanical loading fragments and degrades elastin and is replaced by much stiffer collagen giving rise to decreased wall distensibility [1]. Aortic stiffness can be assessed via quantification of pulse-wave velocity (PWV), which is defined as the rate at which blood motion is transmitted. It is typically quantified by measuring the time delay of the systolic pressure wave at some downstream location, using pressure transducers [2,3] or Doppler US [4] placed at the two locations (typically carotid and common femoral arteries). The method has significant limitations in that the actual path length of the wave is not known. Further, arterial tortuosity increases with age and can vary from subject to subject [5]. By contrast, cardiovascular magnetic resonance (CMR) allows an accurate measurement of the path length and provides regional differences in aortic stiffness [6-9]. Cardiac-gated phase contrast CMR in the sagittal plane [8,10], "pencil beam" excitation [11,12] or sinusoidal tags of a column of blood [13] has been be used to evaluate the thoraco-abdominal aorta but implementing these methods for the peripheral arteries may not be feasible due to increased tortuosity and smaller diameter. On the other hand, cardiac-gated phase contrast CMR in the axial plane [14] can be extended to the peripheral arteries since the pressure pulse transit time will increase distally with respect to the cardiac trigger.

Unlike a central artery, there is sparse PWV data on viscoelastic peripheral arteries (e.g. brachial and femoral arteries) that have higher smooth muscle content. In contrast to the aorta, data suggest (based on ultrasound methods) that the distensibility of muscular arteries remains constant with age [15-19]. According to the Moens-Korteweg formula, $PWV=1∕\sqrt{D\rho }$, the age-related effect on the peripheral PWV will be absent, where *D *is the distensibility coefficient [18] and ρ is the density of blood. Quantification of distensibility requires measurement of the change in the artery's diameter between systole and diastole and is related to the frequency independent component of the vessel wall's dynamic modulus [6,20]. On the other hand, the wave-front velocity approach, commonly known as the "foot-to-foot" method, inherently captures the high-frequency component of the dynamic modulus associated with the rapid increase in pressure during early systole. The dynamic modulus of elastic and viscoelastic arteries has different response to oscillatory pressure waves created by pulsatility. For elastic arteries (e.g. carotids and aorta) PWV quantification by both methods is expected to give approximately the same result since there is little or no lag between the changes in lumen diameter in response to a pressure increase. Muscular arteries such as femoral and brachial arteries have much higher smooth muscle content, which confers their viscoelastic properties. The dynamic modulus at 10 Hz can be 3 to 4 times greater than at 1 Hz in young subjects (<20 yrs), whereas the modulus plateaus at 1 Hz in older (> 35 yrs-old) subjects [21]. Thus, the estimation of stiffness or PWV based on distensibility estimation may not be optimum for assessing structural remodeling of muscular arteries associated with aging or endothelial dysfunction.

In this work we extend the previously developed projection-based approach [22] to estimate PWV along the thoraco-abdominal aorta and the iliofemoral arteries by "simultaneously" exciting and collecting velocity-encoded projections at two arterial segments. The method achieves high temporal resolution without gating by sampling the center k-space line repeatedly to approximate the velocity-time curve during the cardiac cycle from a measurement of the complex difference (CD) signal intensity. The goal of this work is to demonstrate the feasibility of the proposed method in healthy subjects.

## Method

### Non-triggered quantification of PWV with velocity-encoded projections

The CD between velocity-encoded projections retains signal from the moving spins only and represents average signal across the lumen in the projection direction (perpendicular to the readout direction). In order to avoid interference from nearby vessels a suitable readout direction must be chosen. At the onset of systole (flow velocity <<*v_max_*) CD intensity is approximately proportional to velocity, thus the wave-front velocity method can be applied to the time-resolved CD curves to estimate the wave-front propagation time. For example, *sin(φ/2) *and φ*/2 *will differ by only 6% even at *v *= 0.5*v_max _*for an encoding velocity (*VENC*) ~1.33*v_max_*.

**Figure 1:**
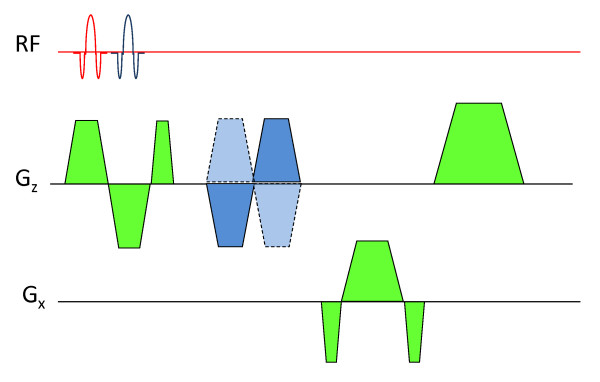
**The pulse sequence for collecting temporally-resolved CD of velocity-encoded projections**. The pulse sequence for collecting temporally-resolved CD of velocity-encoded projections at two arterial segments. The bipolar gradients shown in blue are toggled to encode velocity. The two successive RF pulses differ in carrier frequency so as to excite superior and inferior slice locations.

For the aortic arch only one slice [22] is excited to obtain the CD signal at two arterial segments (proximal ascending and distal descending aorta). For "straight" arterial segments (e.g. thoraco-abdominal and iliofemoral arteries), the CD signal is acquired at two arterial sites quasi simultaneously following two successive excitations via two RF pulses of different carrier frequency in the presence of slice-selective gradients (Figure 1). The polarity of the slice-selective gradient (also serves as a rephasing gradient for the 1^st ^RF) of the 2^nd ^RF is reversed to minimize TR and velocity encoding is implemented with bipolar gradients. The slice separation distance ranges from approximately 250 mm (thoraco-abdominal) to 375 mm (iliofemoral), which corresponds to resonance offset of 213 to 320 kHz, respectively, at gradient amplitude of 20 mT/m. One microsecond RF time-steps are used to avoid aliasing of the proximal slice into regions near the distal slice and vice versa, i.e. the first aliased plane will be over one meter away with 20 mT/m gradient amplitude. Further, the receive channels of the coils located intermediate between the slices of interest are manually disabled to minimize cross-talk. Thus specific channels are "assigned" to a particular slice and the data are reconstructed independently.

### Subjects

Written informed consent was obtained prior to all examinations following an institutional review board-approved protocol. Ten healthy subjects (N = 10, age range 23 - 68 yrs, mean age 44 ± 14 yrs) without prior history of cardiovascular disease were recruited to evaluate the feasibility of quantifying PWV from aortic arch to iliofemoral arteries.

### CMR Protocol

#### Aortic arch

All studies were performed at 3T system (Siemens Tim Trio) using two body matrix and spine coils. The body matrix coil is a 6-element design with 2 clusters of 3 elements, designed so that each cluster can be manually turned on or off. The elements within each cluster are arranged laterally across the chest or abdomen so that each cluster is sensitive to different region along the head-foot direction. An oblique sagittal image through the aorta is first acquired to prescribe and generate multiple axial slices below the pulmonary trunk. Once a suitable axial slice is selected an appropriate readout direction is chosen to avoid vessel interference in velocity-encoded projections, as described in [22]. The imaging parameters for the quantification of PWV along the aortic arch are similar to the previous studies: FOV = 448 mm, voxel size = 2 ×10 mm^2^, TE/TR = 2.6/5.0 ms, bandwidth = 893 Hz/pixel, flip angle = 15° and *VENC *= 175 cm/s (corresponding to 350 cm/s for complex difference). After collecting a reference image (for the purpose of identifying vessels in CD images) 1024 pairs of velocity-encoded projections are acquired in free-breathing mode, covering about 10-12 heart-beats at a temporal resolution of 10 ms. The data acquisition time is under 12 s.

#### Thoraco-abdominal aorta

Body matrix coils are placed on the chest and abdomen such that the proximal descending (Da) and abdominal aorta (Aba) proximal to the iliac bifurcation are approximately centered about a cluster of each body coil. Axial scout images are acquired over the maximum FOV allowed (400 mm) with 20 mm gap to prescribe the two desired slice locations typically separated by 200 to 300 mm. Lastly, the midpoint (halfway between the two prescribed slices) is repositioned to the isocenter of the scanner. The imaging parameters are as follows: FOV = 352 mm, voxel size = 1.38 × 10 mm^2^, TE/TR = 4.0/6.0 ms, bandwidth = 781 Hz/pixel, flip angle = 15^o ^and *VENC *= 125 cm/s. The increased TR (relative to the one used for the aortic arch) caused by lower *VENC *results in a temporal resolution of 12 ms but compensated more than enough from the increase in the path length from about 120 mm to about 280 mm for thoracoabdominal aorta. Even though the temporal resolution is reduced by 20% the path length is more than doubled on average thus leading to higher precision compared to measurements at the aortic arch.

#### Iliofemoral arteries

**Figure 2:**
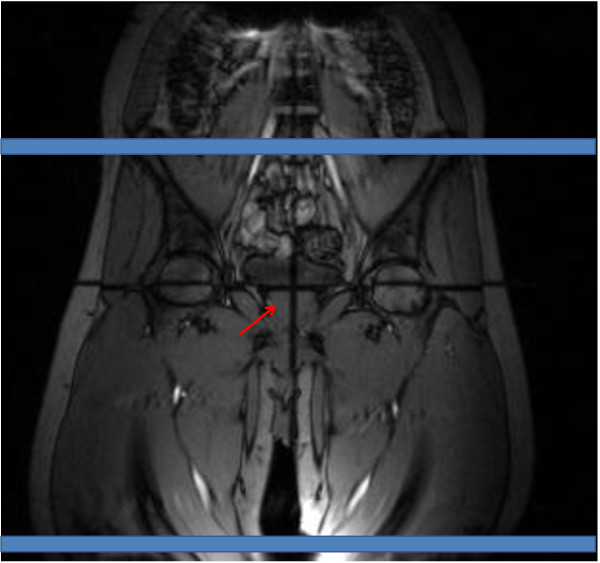
**Coronal image showing prescribed slices**. Coronal image showing prescribed slices (blue bars). The black cross hair (red arrow) marks the isocenter.

Subsequently, the body matrix coil is repositioned from the chest to the thigh. The procedure follows the one above to prescribe the arterial segments of interest: distal to the aortoiliac bifurcation and femoral artery, separated by approximately 350 to 400 mm are prescribed (Figure 2). All imaging parameters except for *VENC *(80 cm/s) are identical to those used for the thoraco-abdominal protocol. Even though the peripheral pulse wave travels faster the precision is still higher because the path length is increased by three to four-fold. The average total scan session time (including the set-up) was less than 30 mins.

### Data Analysis

**Figure 3:**
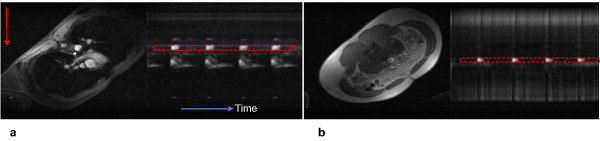
**Reference image and corresponding projection CD images**. Reference image and corresponding projection CD images of Da (**a**) and Aba (**b**) at the level of pulmonary artery and approximately one cm above the bifurcation, respectively. Each column of pixels represent a time point and represents a CD signal from two projections; approximately 400 successive projections are shown. The readout direction is indicated by the red arrow and the time axis is from left to right as indicated by the blue arrow.

**Figure 4:**
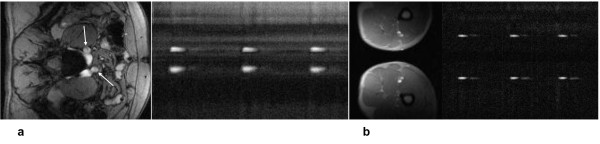
**Reference image and corresponding CD projections**. Reference image and corresponding CD projections of iliac arteries inferior to the bifurcation (**a**) and at the level of the femoral arteries (**b**).

The time-resolved CD signals (spatially averaged along the readout direction) from proximal and distal arterial segments are used to estimate the propagation time of the wave-front as described previously [22]. In general complex difference does not completely remove the static tissue signal (c.f. Figures 3 and 4) resulting in a small relative offset between the two CD time-curves. We use the end-diastolic segment of CD time-curves to determine the magnitude of the offset because the blood flow velocity (which is directly proportional to the CD signal) is nearly zero or equal at both arterial segments. After the offset correction the wave-front propagation time is estimated as an average of temporal separation approximately at the lower-third of the upslope. This is performed for each heart beat and averaged over the 8 to 10 cardiac cycles. The transit time estimation described is equivalent to the foot-to-foot technique that is commonly utilized in tonometric studies [23]. In short, the foot of the curve is determined with the linear regression of the initial systolic velocity wave upslope from the velocity values between 10% and 30%. As a result of the near-linearity of the upslope is linear the systemic error from this approach should be negligible after averaging over several cardiac cycles.

The path length *L *of the wave-front is estimated from the oblique sagittal (aortic arch) and axial scout images (thoraco-abdominal and iliofemoral arteries) acquired previously for prescribing slices of interest. On the sagittal image a center-line of the aorta between the two aortic sites is manually drawn to estimate the path length. In the axial images, the coordinates of the artery's centroid at each slice is recorded to compute the in-plane displacements (Δ*x*, Δ*y*) of the vessel's centroid, where adjacent slices are separated by cm. Thus the path length is given by $L=\sum_{i=1}^{N-1}{\left(\sqrt{\Delta x^{2}+\Delta y^{2}+\Delta z^{2}}\right)}_{i}$, where *N *is the number of axial slices. The PWV is then calculated as$L∕\bar{\Delta t}$, where $\bar{\Delta t}$ is the propagation time averaged over multiple heart beats.

## Results

**Figure 5:**
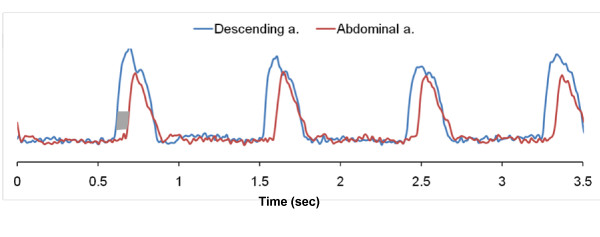
**Temporally resolved |CD| in proximal descending and abdominal aorta**. Temporally resolved |CD| in proximal descending and abdominal aorta of **Fig 3**. The temporal "lag" of abdominal aorta CD signal is apparent. The average temporal separation in the gray shaded region is computed to yield the transit time of the wave-front.

**Figure 6:**
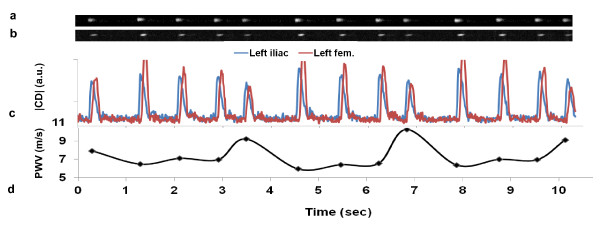
**Magnified view of time-resolved CD image**. Magnified view of time-resolved CD image of left iliac (**a**) and femoral artery (**b**) in a subject with PVCs (occurring at approximately t = 3.5s and 7s). **c**) Plot of average CD signal across the lumens of iliac and femoral arteries. **d**) Apparent elevated PWV corresponds to heart cycles during which a PVC occurred.

Representative magnitude and complex-difference projection images of the proximal Da and Aba are shown in Figure 3. There is no trace of aliasing of the proximal into the distal slice and vice versa on either the magnitude image, and on the CD images Da and Aba can be clearly identified. Similarly, Figure 4 shows magnitude and CD images of proximal iliac and femoral arteries. Plots of the temporally resolved CD signal of the data in Figure 3 are displayed in Figure 5. Figures 6a and 6b are the time-resolved CD images of iliac and femoral arteries, respectively, from a young subject with premature ventricular contraction (PVC). The corresponding CD time-curves are shown in Figure 6c. The non-triggered projection approach avoids gating errors arising from heart rate irregularities. PVCs result in elevated PWV (Figure 6d) presumably due to incomplete relaxation of the aorta.

**Figure 7:**
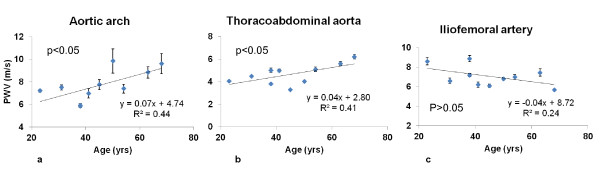
**PWV vs. age of three arterial segments**. PWV vs. age of three arterial segments spanning aortic arch to femoral arteries at mid-thigh. Error bars represent standard error of PWV averaged over multiple heartbeats.

In the PWV versus age plots (Figure 7) the age-related increase in the PWV was significant in the aortic arch (Figure 7a, p < 0.05) and thoracoabdominal aorta (Figure 7b, p < 0.05) with values in good agreement with those quantified previously [7,8,24,25]. The results for the thoraco-abdominal PWV (Figure 7b) agree qualitatively with previous studies [8,21], where the age-related effect in PWV was found to be reduced compared to the aortic arch. Lastly, the data suggest PWV in the iliofemoral arteries to decrease with age (Figure 7c) as observed by Learoyd et al [21], an effect ascribed to the weakening of the muscular arteries as a result of age-related loss of smooth-muscle content [26].

## Discussion and Conclusions

This work demonstrates the feasibility of quantifying three regional (aortic arch, thoraco-abdominal aorta and iliofemoral arteries) PWVs by collecting velocity-encoded projections repeatedly at arterial segments of interest. The proposed non-triggered method is less sensitive to patient motion and irregular heart rhythm as PWV is estimated in "real-time" for each heartbeat. Further, the results suggest that the method may be able to capture the age-related increase in the stiffness of the aortic arch and to distinguish differences in the aging process of elastic and muscular arteries. Epidemiological and autopsy studies previously demonstrated a close link between aging and symptoms of atherosclerosis [26]. Thus, the ability to quantify the effect of age on peripheral PWV in conjunction with the behavior of a central artery may provide additional insight into systemic disease. To the best of our knowledge, peripheral PWV has not been quantified using CMR and the transducer approach has primarily targeted the superficial brachial artery. The present study was designed to demonstrate feasibility and was not powered to evaluate the dependence of age on the two different types of arteries.

We observed variation in PWV from one cardiac cycle to another as indicated by the average standard error of about 0.3 m/s (c.f. Figure 7). A few studies have reported heart rate dependence of PWV [27-29] but, to the best of our knowledge, studies involving variations in PWV between cardiac cycles have not previously been reported. The variation in R-R interval at rest is not uncommon and can lead to a different relaxation state of the artery, thus variations in PWV between successive cardiac cycles are expected. Unfortunately, intersubject standard deviations instead of individual standard error are typically reported in the literature even though PWV estimated via tonometry, CMR or Doppler is derived from many cardiac cycles. The heart rate variation cannot fully explain the differences in the PWV between different cardiac cycles. However, we observed that the standard error is smaller in the thoracoabdominal aorta and iliofemoral artery compared to the aortic arch. This is not surprising since the separation distance between the proximal and distal arterial segments are much larger (250 to 350 mm in the thoracoabdominal aorta and iliofemoral artery segments compared to about 120 mm in aortic arch), resulting smaller error in transit time estimation. However, the overall error is reduced by averaging the PWV over 8-10 cardiac cycles as demonstrated previously [22] in which the projection method was compared directly against the established cine PC-CMR.

The method is limited to slices where vessel interference can be avoided with an appropriate choice of readout orientation. This limitation is exacerbated when projections have to be acquired simultaneously at two slices thus requiring careful planning for prescribing the slices of interest. In the peripheral arteries, vessel overlap with the veins could be ignored considering that the venous CD signal was at the level of noise (c.f. Figures 4a and 4b). We implemented two successive RF pulses instead of modulating the *sinc *pulse by a cosine function to reduce average and peak SAR. However, the two RF pulses were separated by less than one millisecond and thus the decrease in the CD signal due to relaxation and blood flow motion is negligible.

For elastic arteries such as the aorta, PWV can be quantified with either the foot-to-foot approach or by estimating distensibility. In fact, recent study by Dogui et al [6] showed consistency between the aortic distensibility and PWV with respect to the Bramwell-Hill model [30]. However, the two methods are expected to disagree on muscular arteries (e.g. femoral artery) that are better characterized as viscoelastic material where the modulus of the arterial wall has stronger dependency on the oscillatory nature of the arterial pressure fluctuation. Since measurement of distensibility fails to capture the age-related effect (possibly endothelial dysfunction, as well) we suggest the wave-front velocity based method to be better suited for assessing pathophysiology of vascular aging and atherosclerosis of muscular arteries. However, addressing these questions will require comparative studies in larger cohorts of healthy subjects and patients with subclinical atherosclerotic disease.

## Competing interests

The authors declare that they have no competing interests.

## Authors' contributions

ML and CL conceived, designed and implemented the pulse sequence, collected and analyzed the data. FW participated in the design of experiment and study, and helped to draft the manuscript. All authors read and approved the final manuscript.
